# Factors associated with patients’ understanding of their management plan in Tshwane clinics

**DOI:** 10.4102/phcfm.v6i1.560

**Published:** 2014-01-31

**Authors:** Leticia Fernandez, Theresa Rossouw, Tessa Marcus, Angelika Reinbrech-Schutte, Nicoleen Smit, Hans F. Kinkel, Shehla Memon, Jannie Hugo

**Affiliations:** 1 Department of Family Medicine, University of Pretoria, South Africa; 2 Foundation for Professional Development, South Africa

## Abstract

**Background:**

This research focused on patients’ views regarding healthcare services and identified factors associated with understanding of their management plan.

**Aim:**

To develop a baseline for patient–clinician collaboration and the extent to which patients felt included and understood their treatment plan.

**Setting:**

Tshwane district (South Africa) public health outpatient clinics.

**Method:**

Medical students interviewed 447 patients in 22 clinics in Tshwane district. Agreement was measured by the percentage of cases in which patients and clinicians were in accord about a particular aspect of the consultation.

**Results:**

About one-third of patients incorrectly answered questions on whether changes in lifestyle or diet were prescribed as part of their treatment. The likelihood that patients understood their plan was associated with seeing the same clinician three or more times; having a consultation in their same or a similar language; patient participation in the diagnosis; and feeling that the clinician had explained their health problems to them.

**Conclusions:**

There is need for greater emphasis on continuity of care, the clinicians’ ability to speak the patient's language and involving patients in the consultation.

## Abstrait

**Les facteurs associés à la compréhension par les patients de leurs plans de prise en charge dans des centres médicaux de Tshwane**

**Présentation:**

Cette étude se concentrait sur les points de vue des patients concernant les services de santé et les facteurs identifiés associés à la compréhension de leur plan de prise en charge.

**Objectif:**

Développer une référence pour la collaboration entre patients et médecins et déterminer la mesure dans laquelle les patients avaient le sentiment d’être inclus à la préparation de leur plan de prise en charge et de le comprendre.

**Cadre:**

Centres médicaux publics accueillant des patients en consultation externe dans le district de Tshwane (Afrique du Sud).

**Méthode:**

Des étudiants en médecine ont interrogé 447 patients dans 22 centres médicaux dans le district de Tshwane. La concordance était mesurée par le pourcentage de cas dans lesquels les patients et les médecins étaient d'accord sur un aspect particulier de la consultation.

**Résultats:**

Environ un tiers des patients n'a pas su répondre correctement aux questions visant à déterminer si des changements dans leur style de vie ou régime alimentaire avaient été prescrits dans le cadre de leur traitement. La probabilité que les patients comprennent leur plan était associée au fait de consulter le même médecin à trois reprises ou plus, de consulter dans leur langue ou dans une langue similaire, de participer au diagnostic, et au sentiment que le médecin leur avait expliqué le problème de santé dont ils souffraient.

**Conclusions:**

Il est nécessaire de mettre davantage l'accent sur la continuité de la prise en charge, la capacité des médecins à parler la langue de leurs patients, et l'implication des patients dans la consultation.

## Introduction

South Africa is a multicultural, multilingual society where there is extreme social inequality and extensive racial inequity that manifest both in health outcomes and access to quality healthcare. For the majority of the population, historically-created racialised endemic poverty and social dislocation have produced substantial health and education deficits over generations.^1, 2^ The resultant disease burden has been further compounded by the HIV and tuberculosis (TB) epidemics, which threaten to overwhelm an already-stretched public healthcare delivery system.^3^

In the presence of fragmented health systems, low levels of health literacy, severe shortages of staff and medical professionals and poor staff morale, quality of care in public clinics and hospitals has remained low for the most vulnerable groups in South Africa.^1, 3, 4^ A recent study to evaluate the quality of services in 16 HIV clinics in and around Pretoria that offer antiretroviral treatment (ART) found that they underperformed in critical areas when measured against performance standards for ART initiation. Clinician consultations were amongst the weakest areas in terms of quality of service. The study found that psychosocial histories were taken from only 50% of the patients, physical examinations were performed in only 41% of these consultations and only 38% of patients were asked to bring all concurrent medications to their next visit. In the case of female patients, only 56% were asked if they were pregnant and only 39% were referred for a pap smear. Moreover, the authors report that longer consultations were not necessarily associated with higher quality of clinician care.^5^

In the next decades, improving the quality of healthcare and health outcomes may well depend on changes in the delivery of care, such as promoting patient-centred care that will contribute to better relationships and more effective collaboration between patients and clinicians.^4, 7, 8^ Patient-centred care improves outcomes because it is informed by patient understanding of the issues and their treatment preferences, as well as their interest and commitment to managing their own health.^7–9^ In this sense, the caregiver acts as an interpreter of the patients’ symptoms and health needs and engages with them as partners in the quest to improve their long-term health prospects.^10^

The positive impact of patient-centred care has been documented in several previous studies on emotional, mental and functional health, treatment adherence, physiological status (e.g. blood pressure), symptoms resolution and pain control.^11, 12^ Specific benefits associated with positive patient–clinician communication include a reduction in patient distress and blood pressure when clinicians provide clear information and emotional support, as well as diminished patient anxiety when clinicians encourage questions and share in the decision-making process.^11^ Patient participation during consultation and agreement with the clinician about causes and treatment for their symptoms are significantly associated with better medication adherence and clinical outcomes.^8, 12^

Amongst the current barriers to effective patient-centred practices in public clinics and hospitals in South Africa are a lack of continuity of care, time constraints, limited resources and language and cultural differences.^13–15^ Continuity of care is especially important in patient-centred care because it creates the opportunity for developing a therapeutic relationship between patients and clinicians over time.

The concept of continuity of care has evolved with changes in the context of healthcare delivery over time. In the 1950s, when single-physician practices were the prevailing mode, continuity of care referred to having a personal caregiver. As the number of medical partnerships increased, concerns about patient anonymity led to the redefinition of continuity of care as coordination amongst caregivers to ensure uninterrupted patient care. Later on, the restructuring of the healthcare systems of the 1990s resulted in redefining continuity of care to include not only continuity by the same caregiver or group of caregivers, but also coordination of services along the components of the care delivery system.^16^

More recently, the hierarchical model of continuity portrays a continuum of care from informational to longitudinal to interpersonal continuity. At the lowest level, informational continuity refers to a group of clinicians having access to comprehensive knowledge about a patient without necessarily developing a relationship. Longitudinal continuity refers to patients receiving care from a team of clinicians who are responsible for coordinating healthcare services. At the highest level, interpersonal continuity involves a relationship in which the patient develops trust in a clinician, who then assumes responsibility for the patient's overall healthcare.^17^

Throughout the changes in the concept of continuity of care, however, the one constant has been that it represents the patient's perspective. The three major themes have been the importance of a personal caregiver committed to the patient's wellbeing, communication of patient information between caregivers and coordination between caregivers to ensure uninterrupted care.^16^ Basically, continuity of care is about the way that individuals experience and practitioners provide healthcare over time.^6^ Continuity of care happens when healthcare is connected, coherent and consistent.^18^ Continuity of care improves the quality of care with clinicians enjoying greater patient trust. It also contributes to improved health outcomes with, amongst other things, patients being more likely to follow advice, having fewer and shorter hospitalisations and making less use of emergency services.^19–27^

Clinician cultural and linguistic competences are also important, especially in patient-centred care, since they aim to bridge cultural and socioeconomic differences, improve communication and develop a meaningful patient–clinician relationship. Clearly, when clinicians are unable to understand their patients’ complaints or needs and patients, in turn, do not understand the advice of the clinician, quality of care is compromised.^28, 29^

We report findings from a quality-improvement project conducted in Tshwane clinics to develop a baseline for patient–clinician collaboration during consultation. The project is part of the service-learning opportunities designed for medical students by the University of Pretoria's Departments of Family Medicine and Public Health. This study measured the extent to which patients report that clinicians adhere to standards of performance during consultation; the relationship between patient–clinician collaboration on patient understanding of management plans; and whether other factors, such as language and continuity of care, mediate the information exchanged between patient and clinician.

## Research methods and design

The authors developed the research objectives, protocols and instruments to establish a baseline for patient-clinician collaboration during consultations in Tshwane's public health clinics. Between one and four Year 5 medical students from the University of Pretoria were assigned to each of the 22 clinics that participated in the study, depending on the facility size and its ability to accommodate students.

The questionnaires for patients and clinicians were developed in English, mirrored each other and contained several statements about the consultation. The language of the interview with patients was expected to be English, but students reported that if they felt it was needed they used Afrikaans or some other African languages they knew. However, a limitation of this study is that we did not record the language or languages of the interview, neither did we measure the language proficiencies of the interviewers and respondents. (See appendix 1 for sample questionnaire)

Statements about the consultation could be answered as ‘true’, ‘partly true’, ‘partly false’ or ‘false’. The answer categories for statements about the patient's management plan were ‘yes’, ‘no’ or ‘not sure’. For example, patients were asked to evaluate the statement, ‘*The clinician listened to my questions and concerns without interrupting me*’. The patient's answer was compared to the clinician's response to a similar statement, namely, ‘*I listened to this patient's questions and concerns without interrupting*’. In addition, data were collected about the patient's age, gender, first language, language of consultation and the number of times the patient had seen the same clinician.

Patients were selected as a sample of convenience and their names and other identifying information were not collected or entered in the database. The only link between the patient and clinician was the patient's file number, which students recorded in both the patient and the clinician questionnaires. Each student interviewed 10 patients post-consultation. The clinician was asked to complete the corresponding form on their own immediately after the consultation to ensure that the interaction would refer to the particular patient interviewed. After interviewing a patient, the student collected the completed questionnaire from the clinician and matched them by the patient's file number. Clinicians and patients were blinded to each other's answers, which students were instructed to treat as confidential information. Students entered the matched patient–clinician data into a database prepared by the authors for this study.

In this paper we present the combined findings from all the clinics in the research project. In total, there were 447 paired patient–clinician observations. However, one variable, the number of visits to the same clinician, had 78 missing cases (17.5%), which reduced the usable dataset to 366 pairs of observations. Preliminary analysis showed that the cases with missing data did not differ significantly in terms of patients’ age, gender or language from those with no missing data. In order to address the missing values in the dataset and maximise the sample size, we used the imputing function of Stata, using as regressors all other patient variables in the survey to inform the imputation of values for the number of visits. No imputations were done for any other variable. We present our findings using the larger dataset with imputed values. Findings from the analysis with the smaller data set with no imputations are similar, except that the association between number of visits to the same clinician and the dependent variables do not reach statistical significance using the smaller dataset with no imputed values, even though the direction of the coefficients are similar.

### Data analysis

The two outcomes of interest are whether patients gave the same answer as their clinician regarding the statements (a) ‘*My treatment or management plan includes changes in lifestyle*’, and (b) ‘*My treatment or management plan includes changes in diet*’. We coded each of these variables as 1 if both the patient and the clinician answered the same (‘yes’, ‘no’ or ‘not sure’) and coded 0 otherwise. We hypothesised that patients would be more aware of these details of the management plan when they experienced greater collaboration during consultation, have continuity of care and/or the consultation is in their first or a similar language.

The independent or explanatory variables central to the analysis are:

(1) **Continuity of care:** The number of visits with the same clinician was obtained from patients. This variable is coded 1 if the patient had met the same clinician three or more times and 0 if the patient saw the clinician for the first or second time. The choice of three or more consultations as a cut-off point was based on empirical comparisons. Prior to imputation, the percentage of respondents who matched their clinicians’ responses about whether their management plan included changes in lifestyle was 70.6% for those seeing the clinician for the first time (*n* = 197); 71.7% if they had seen the same clinician for the second time (*n* = 53); and 79.4% amongst those seeing the clinician for the third time (*n* = 34). These differences became of statistical significance after the imputation of missing values for the number of times seeing the same clinician.

(2) **Language concordance:** This variable is coded as 1 if the consultation was conducted in the patient's first language or a same or similar language; and coded 0 if the language of consultation is different from the first language of the patient. This variable takes into account that some languages are related, such that speaking one facilitates understanding and communicating in the other. For example, IsiZulu, IsiXhosa and Xitsonga are considered similar languages, as are Sepedi, Sesotho and Setswana.

(3) **Consultation elements:** The statements included in the analysis were selected based on two criteria: significant bivariate correlation with the dependent variables and/or their conceptual relevance to patient-centred care. Each variable is coded as 1 if both the patient and clinician answered ‘true’ or ‘partly true’ and 0 otherwise.

The next section presents the sample characteristics, the extent of agreement between patients and clinicians and the results of multivariate analysis. Because of the dichotomous nature of our dependent variables, we use logistic regressions to assess the role of continuity of care, language concordance and selected consultation elements on patients’ understanding of their management plan.

### Ethical considerations

This study was approved by The University of Pretoria's Faculty of Health Sciences Research Ethics Committee (Protocol #75/2010). Prior to data collection, students received training about the purpose of the study and the use of protocols to recruit and interview patients. Students also attended a second session to address any issues related to the study. There were no anticipated risks associated with participation in the study for either the patients or the clinicians and the potential benefits to the patients included improvement in clinician understanding of patient perspectives regarding medical services. Patients recruited for the study were informed that their participation was voluntary and that their answers would be strictly confidential and anonymous, with no negative consequences in terms of treatment at the clinic based on their answers or if they chose not to participate.

## Results

The characteristics of patients interviewed in the participating Tshwane district clinics are listed in Table 1. Most of the patients interviewed were female (66%; *n* = 293) and over half (52%; *n* = 232) were aged 45 or older, with 12% (*n* = 51) aged 65 or older. Since patients in the clinics are assigned to the next available clinician, 48% (*n* = 213) of the patients consulted with a clinician that they were meeting for the first time, 13% (*n* = 59) had seen their clinician once before and 39% (*n* = 171) had seen their clinician three or more times.

**TABLE 1:** Sample characteristics: patients interviewed in Tshwane District clinics.

Variables	Patients (*n* = 443)	%
**Gender**		
Female	293	66.1
Male	150	33.9
**Age group**		
< 25	46	10.4
25–44	165	37.2
45–54	101	22.8
55–64	80	18.1
65 +	51	11.5
**Consultations with clinician**		
First time with this clinician	213	48.1
Second time with this clinician	59	13.3
Three or more times with this clinician	171	38.6
**Language of consultation and patient's first language**		
*Patients whose consultation was in a language different*		
*from their first language*	238	53.7
English	186	42.2
Afrikaans	36	8.1
Sepedi/Sesotho/Setswana	14	2.9
Isizulu	2	0.5
*Patients whose consultation was in their same or similar language*	205	46.3
Sepedi/Sesotho/Setswana	74	16.7
Afrikaans	68	15.3
English	31	7.0
Isizulu/Isixhosa/Isindebele	26	5.9
Other	6	1.4

Table 1 shows that there is a large diversity of languages spoken at the clinics by both patients and clinicians. Fifty-four per cent (*n* = 238) of the patients who had a consultation in a language other than their first language, had this consultation in English (42%; *n* = 186) or Afrikaans (8%; *n* = 36). On the other hand, close to half the patients in the sample (46%; *n* = 205) had a consultation in their own or a similar language, mostly in the related languages of Sepedi/Sesotho/Setswana (17%; *n* = 74) and in Afrikaans (15%; *n* = 68).

Table 2 shows several of the statements that patients and clinicians were asked to evaluate independently after the consultation. Most patients and clinicians answered that these statements were true or partly true about their consultation, resulting in very high agreement.

**TABLE 2:** Agreement in patient–clinician matched items (*n* = 443).

Questions to patients	Questions to clinicians	Percentage matched answers†
The clinician listened to my questions and concerns without interrupting me.	I listened to this patient's questions and concerns without interrupting.	98.4
The clinician examined me in order to find out what's wrong with me.	I examined this patient in order to find out what's wrong with her or him.	92.1
I participated in the diagnosis of my condition or disease.	This patient participated in the diagnosis of her or his condition or disease.	87.8
The clinician explained to me what's wrong with my health.	I explained to this patient what's wrong with her or his health.	95.5
I had the opportunity to ask questions of the clinician.	I encouraged this patient to ask me questions about her or his condition or disease.	92.8
I feel confident I can explain to someone at home the treatment or management plan for my condition.	This patient will be able to explain to someone at home the management plan forher/his condition.	95.5

† Matched answers are those where clinician and patient answered that the statement was true or partly true.

Table 3 compares answers from clinicians and patients regarding the management plan. The first two statements, whether the patient has the same understanding as her or his clinician regarding the need for changes in lifestyle and changes in diet as part of the management plan, are the outcome variables in this analysis. Approximately 67% (*n* = 296) of the clinicians said that the management plan of the patient they had just consulted included changes in lifestyle, 32% (*n* = 142) answered that it did not and 1% (*n* = 5) said they were not sure. It is noteworthy that the few cases (*n* = 5; 1%) in which clinicians answered that they were ‘not sure’ about the changes in their patient's management plan usually referred to seeing a patient for the first time and not finding sufficient information about their management plan on the patient's records. This issue is complex and will not be discussed for now as it is outside the scope of this study.

**TABLE 3:** Clinicians’ vs. patients’ understanding of their management plan.

Statements about the patient's management plan	Clinicians’ answers		Patients’ answers		Matched clinician-patient agreement†	
		
	*n*	%	*n*	%	*n*	%
**The treatment or management plan includes changes in lifestyle (** ***n*** **= 443)**						
Yes	260	58.7	268	60.5	229	51.7
No	142	32.1	155	35.0	97	21.9
Not Sure	5	1.1	20	4.5	1	0.2
**Total matching,%**	-	-	-	-	-	**73.8**
**The treatment or management plan includes changes in diet (** ***n*** **= 443)**						
Yes	260	58.7	242	54.6	201	45.4
No	183	41.3	189	42.7	138	31.2
Not Sure	0	0.0	12	2.7	0	0.0
**Total matching,%**	-	-	242	54.6	-	**76.5**
**The treatment or management plan includes seeing another doctor, specialist or therapist (** ***n*** **= 442)**						
Yes	89	20.1	97	21.9	64	14.5
No	348	78.7	335	75.8	308	69.7
Not Sure	5	1.1	10	2.3	0	0.0
**Total matching,%**	-	-	-	-	-	**84.2**

† Clinician and patient answered the same (Yes, No or Not Sure). Non-matching responses are not included in the last column.

Patients’ answers regarding the need for changes in lifestyle as part of their management plan had a similar distribution: 61% (*n* = 268) said ‘yes’, 35% (*n* = 155) said ‘no’ and close to 5% (*n* = 20) were ‘not sure’. Matching the answers between clinicians and their patients in the last column shows that approximately 74% (*n* = 327) gave the same answer, with most agreeing that changes in lifestyle were necessary (52%; *n* = 229). Table 3 also shows that 77% (*n* = 339) of the patients matched their clinician's responses regarding changes in diet and 84% (*n* = 372) matched their clinician's responses about the need to see another doctor, specialist or therapist.

Table 4 shows the odds of patient–clinician agreement regarding whether changes in lifestyle and diet are part of the patient's management plan. The dependent variable is the odds of patients correctly answering whether their management plan includes changes in lifestyle or changes in diet.

**TABLE 4:** Odds ratios of patient–clinician agreement about the management plan.

Variables	Patient–clinician agree† as to whether management plan includes changes in:	

	Lifestyle‡	Diet‡
**Patient's age**	1.00	1.00
**Gender**		
Male	-	-
Female	0.80	1.27
**Times consulting with same clinician**		
One or two time	-	-
Three or more times	1.67**	1.02
**Language of consultation**		
Different from patient's first language	-	-
Same or similar to patient's first language	1.83**	1.60**
**Matched statements about the consultation**		
*The patient participated in the diagnosis of her or his condition*		
Patient and clinician disagree	-	-
Patient and clinician agree	2.40**	1.78*
*The clinician explained the health problem to the patient*		
Patient and clinician disagreed	-	-
Patient and clinician agreed	2.06	2.71**
*The patient had opportunity to ask questions*		
Patient and clinician disagreed	-	-
Patient and clinician agreed	1.20	0.94
*The patient understands what's wrong with her or his health*		
Patient and clinician disagreed	-	-
Patient and clinician agreed	2.26	0.53
**Number of observations**	441	441

† Clinician and patient answered the same (Yes, No or Not Sure).

‡ Disagree is the omitted category.

* Coefficients statistically significant at *p* < = 0.10;

** *p* < = 0.05.

The most important findings from the multivariate analysis is that the odds of patients understanding whether their management plan includes changes in lifestyle are about 70% – 80% higher if they had seen the same clinician more than twice or if their consultation was in their first (or a similar) language compared with those whose consultation was in a different language. Similarly, the odds of correctly answering whether the management plan includes changes in diet were 60% higher for patients consulting in their first or a similar language than for those consulting in a different language.

Table 4 shows that, in addition to the impact of language and continuity of care on better patient understanding of their management plan, patients who agreed with their clinicians that they participated in their diagnosis were about twice as likely to answer correctly whether their management plans included changes in lifestyle or diet, respectively, compared with patients who said they did not participate in the diagnosis. Similarly, when patients agreed with their clinician that their health problem had been explained to them, they were substantially more likely to answer correctly whether or not their management plan included changes in diet.

## Discussion

Given the low quality of care in public South African clinics, it was surprising to find that patients consistently gave very positive assessments about their clinician, as is shown in Table 2. The vast majority of patients interviewed agreed that during their consultation the clinician listened to their questions and concerns, conducted a physical examination, gave them a chance to ask questions, gave them an explanation for their health problems and allowed them to participate in the diagnosis. In addition, nearly all patients agreed that they were informed well enough to explain their treatment or management plan to others.

Even with the seemingly small variation in answers, in preliminary analysis we found that three statements had a statistically-significant association with whether or not patients understood their management plan (*p* < 0.05). These statements were (1) whether the patient participated in the diagnosis, (2) whether the clinician explained the health problem to the patient and (3) whether the patient understood what was wrong with his or her health.

However, the clear bias toward positive assessment of clinicians by their patients warrants future research to understand the limitations of these items. Students suggested two possible reasons: (1) that patients may have been concerned about the confidentiality of their answers, even if assured otherwise and (2) that patients may have little background or other experiences that would help them assess whether their consultation was thorough or not.

We found greater disagreement in comparing the answers of patients and clinicians regarding the content of the management plan (Table 3), which may be because these items refer to factual knowledge. Patients in many cases were not sure or did not know whether their management plans included changes in lifestyle or diet, or referrals to other doctors. This suggests that the exchange of information during the consultation was not understood fully in the same way by patients and clinicians.

An issue with potentially-significant consequences for health outcomes is the relatively high proportion of patients who do not know whether their management plan includes changes in lifestyle or diet. Immediately after their consultation, about 26% (*n* = 116) of the patients were confused about whether or not they needed to modify their lifestyle and 24% (*n* = 104) did not know if they needed to modify their diets. In fact, one-third of all the patients in the study did not answer correctly one or both items about lifestyle and diet changes prescribed in their management plan.

Our main findings are that patients have a significantly better understanding of their management plan when consultations are conducted in the patient's first or a similar language, with a clinician they have seen several times before. Patients also need to feel involved during the consultation and understand the explanation regarding their condition. This suggests the need for greater emphasis on continuity of care and on addressing obstacles to effective communication, either by improving clinician linguistic competencies or by matching clinician–patient language capabilities to whatever extent possible. These are likely to be cost-effective patient-centred interventions with a potentially large impact on patient health outcomes.

There are three limitations of this study. Firstly, the participants were selected as a sample of convenience and may not be representative of the population presenting to all Tshwane clinics. Secondly, the questionnaires were developed only in English and the English language proficiency of respondents was not measured so that there is no information as to whether statements were understood as intended. Thirdly, we imputed missing values for the variable that measured the number of times that the patient had seen a particular clinician. Although we did not find significant differences between those who answered and the missing cases, our findings should be taken as tentative until further research corroborates them.

## Conclusion

This study contributes to patient-centred research that shows how clinicians, through their communication skills and interactions during consultations, have the potential to exert a positive influence with regard to their patients’ health behaviours and outcomes.

In this study, about one-third of the patients presenting in public clinics answered questions incorrectly on whether changes in lifestyle or diet were prescribed as part of their treatment, even though they had just left the consulting room. Clinicians may not be aware of the confusion their patients are experiencing about their medical advice or treatment. This may be due to the stresses of treating a high volume of patients or the need to keep a focus on making the correct diagnosis.

Patient-centred care argues that consultation should be tailored to address each individual's needs and preferences. The shift requires that clinicians engage in conversations with their patients in collaborative ways. This collaboration has the potential to empower patients to participate actively in health matters and is likely to result in better clinical outcomes.^30, 31^

Clinician linguistic competence appears to be especially important. As in other multi-lingual societies, language in South Africa is a marker for imputed or actual cultural differences. Whereas clinicians invariably speak English and/or Afrikaans, the majority of their patients speak their own and other indigenous languages and only a little English or Afrikaans.^32^ In this way, the communication barriers between patients and clinicians that arise from the structure of a healthcare system that anonymises and depersonalises health services are reinforced perversely by language differences. Researchers have reported that medical students doing clinical rotations in health facilities are often frustrated by their inability to connect to and understand the cultural experiences and social constrains that impact the health outcomes of their patients.^32^ Currently, language barriers are addressed on an *ad hoc* basis in most clinics and hospitals in South Africa, with translation help from a staff member if needed.^29, 32^ However, more research is needed in order to understand the extent to which miscommunication due to language barriers reflects on poor health outcomes.

Engaging patients in the consultation and building positive therapeutic relationships are dynamics that are easier to introduce through continuity of care and in the presence of greater patient–clinician language concordance. Admittedly, continuity of care and matching patients to clinicians who are able to speak the same or a similar language may not be possible or cost-effective in large clinic or hospital settings, where it may require investment in staff training and in the development of strategies to address organisational and structural challenges. However, continuity of care may be more easily implemented in primary care at the community level where some innovative transformations are already taking place. Examples of innovative practices include Lusikisiki public clinics in rural South Africa, where HIV services have been integrated into primary healthcare through decentralisation of services and by task shifting within each clinic to distribute workloads more evenly;^33^ another successful innovation has been the adoption of new technologies (eg. geographic information systems [GIS]) in Hlabisa subdistrict primary healthcare clinics to study and address the relationship between distance to roads and clinics and various health outcomes, including the prevalence of HIV amongst women attending prenatal clinics.^34, 35^

Ultimately, improvements in the health of the most vulnerable segments of South Africa may require systemic commitment to patient-centred practices and making community-level primary care the first and most important part of the healthcare system. It may also require that medical training incorporate curricula enriched by the perspectives that social sciences research and linguistic studies can bring to the understanding of health-impacting behaviours. In this regard, reviews of studies with diverse patient populations find strong evidence that cultural and linguistic competence have positive impacts on health outcomes, including improved adherence to treatment and following recommended changes in lifestyle.^36^

Cultural and linguistic components may include a broad range of practices, from endorsement of influential community members and using materials in the preferred language, to adapting messages to cultural values and having providers or peer educators from the same cultural, ethnic or racial groups. Some components may be more relevant than others, indicating that there is need for further research to identify the specific cultural and linguistic components that may be most effective within particular populations.^37^
